# Precise and Efficient Pointing Control of a 2.5-m-Wide Field Survey Telescope Using ADRC and Nonlinear Disturbance Observer

**DOI:** 10.3390/s23136068

**Published:** 2023-06-30

**Authors:** Yang Liu, Yongting Deng, Hongwen Li, Jianli Wang, Dejun Wang

**Affiliations:** 1 Changchun Institute of Optics, Fine Mechanics and Physics, Chinese Academy of Sciences, Changchun 130033, China; liuyang@ciomp.ac.cn (Y.L.); lihongwen1970@yahoo.com (H.L.);; 2 University of Chinese Academy of Sciences, Beijing 100049, China; 3 College of Communication Engineering, Jilin University, Changchun 130025, China

**Keywords:** 2.5-m-wide field survey telescope, linear active disturbance rejection control, nonlinear disturbance observer, nonlinear tracking-differentiator

## Abstract

Linear active disturbance rejection control (LADRC) has been widely used to improve the tracking accuracy and anti-disturbance performance of telescope servo control under disturbances. However, the linear extended state observer (LESO) is sensitive to noise, and its bandwidth is limited by the resonant frequency of the telescope. To enhance the LARDC’s ability to attenuate disturbances, a novel cascade anti-disturbance structure (NCADS) with LADRC on the outer speed loop and a nonlinear disturbance observer (NDOB) on the inner current loop is proposed. The NDOB compensates for the dominant disturbance through feedforwarding the q-axis current reference, and the LESO compensates for the residual disturbance on the outer speed loop. First, the NCADS is introduced in a three-closed-loop control framework of PMSM. Then, the design method of the controller for each loop and the NDOB are presented, the parameter-tuning method based on bandwidth is demonstrated, and the convergence of the NDOB is proved. Furthermore, to improve the searching and tracking efficiency of wide-field survey telescopes, the nonlinear tracking differentiator (NTD) was modified to plan the transition process of the position loop, which only needs to set the maximum speed and acceleration of the telescope. Finally, simulations and experiments were performed on a 2.5-m-wide field survey telescope. The experimental results verify that the proposed NCADS method has a better anti-disturbance performance and higher tracking precision than the conventional method, and the improved NTD method does not need to tune parameters and achieved a fast and smooth transition process of the position loop.

## 1. Introduction

The direct drive technology of permanent synchronous motors (PMSMs) has been widely applied in ground-based, large-aperture telescopes, including the 8.2 m Subaru telescope in Japan, the 10.4 m GTC telescope in Spain, and the four 8.2 m VLT telescopes in the European Southern Observatory, because of their advantages, such as their high power density and good low-speed performance [1,2]. As capability for deep space exploration and requirements for wide-field surveys grow, the apertures of ground-based telescopes are becoming larger and larger. Consequently, a direct drive PMSM servo system with higher driving capability and tracking accuracy is required. For the main axis servo control of large telescopes, the following major challenges have been confronted in engineering practice:

(i) As the telescope diameter increases, the mechanical resonance frequency decreases, and the control bandwidth becomes narrower. In addition, disturbances caused by cogging torque, nonlinear friction, and random increases in wind load make it difficult for classical PI controls to meet the requirements of high performance, and the final image quality will be bad if the controller does not reject the disturbances adequately.
(ii) Wide-field survey telescopes usually work in a searching and tracking mode; that is, the telescope first points to the target area, takes a long exposure picture, then subsequently points to the next target area, takes another long exposure picture again, and so on. Therefore, fast and smooth pointing without overshooting the target area is crucial to improve the efficiency throughout the whole night. So, the transition process must be planned according to the acceleration capacity.
(iii) Many advanced and complex control algorithms have been developed; however, because their design or parameter-tuning methods are too complicated, they are rarely used as widely as PID in engineering practice. Because of its simple design and easily tuned controller, it is best to not change the classical three-closed-loop control structure, as it is more favorable to engineers.

To handle the problems encountered in telescope servo control, many improved PID control methods have been developed, such as the anti-windup internal PID [3] and variable structure PID [4]. These methods were designed to solve the contradiction between speed and overshooting. However, there is still much room for further improvement in the high-precision tracking and disturbance rejection performance, so a new disturbance-rejection PID was proposed in [5]. The authors of [6] analyzed internal and external disturbances in PMSM drives and summarized disturbance estimation and attenuation techniques. In telescope servo systems, the internal disturbance mainly includes cogging torque and nonlinear friction, and the external disturbance mainly includes wind load.

Han proposed an active disturbance rejection controller (ADRC) [7,8] that included TD, NLSEF, and ESO. ESO is a new concept of estimating the total disturbance without distinguishing between internal and external disturbances. Gao analyzed the control paradigm of ADRC and introduced a linearization method to achieve linear active disturbance rejection control (LADRC), which enabled engineers to tune the LADRC parameters based on the concept of bandwidth [9], significantly promoting the application of ADRC. The authors of [10] applied LADRC to the speed loop control of a small telescope servo system, and LADRC had the same bandwidth as PI. The LADRC effectively suppressed the friction and cogging torque fluctuations; thus, the low-speed tracking precision was improved compared with PI. However, for small telescopes, the resonant frequency is several times higher than that of large telescopes, and the LADRC parameters were set to wo = 500 and wc = 100 in the paper, so the bandwidth of the LESO was hardly limited by the resonant frequency. Moreover, ref. [11] applied LADRC to the speed loop of a 1.2-m telescope. The closed bandwidth of the speed loop was designed to be 13.7 Hz; however, the LADRC parameters were only set to wo = 40 and wc = 70, and the bandwidth of the LESO was obviously limited. It is thus necessary to enhance the disturbance rejection ability of the speed loop to achieve higher tracking precision under limited bandwidths.

An overview of the disturbance-observer-based control (DOBC) method was provided in [12]. In 2020, a survey of the major results from studies over the past 35 years on DOB-based robust control was presented in [13]. Robust control techniques can be divided into two categories: suppressing disturbances via feedback control, such as PID and internal model control (IMC) [14], and canceling disturbances via feedforward control, such as DOBC [15] and ADRC [16]. It was theoretically and experimentally proven that the robustness and performance of a control system can be independently adjusted using a DOB and a performance controller, respectively, which is referred to as the two-degrees-of-freedom (2-DoF) control structure [17]. Some control methods, such as PID and sliding mode control (SMC) [18], have been combined with DOB to apply the 2-DoF control structure to improve the performance of systems. A cascade acceleration feedback control (AFC) enhanced by a disturbance observation and compensation (DOC) method was proposed to improve the tracking precision of telescope systems under disturbances [19]. However, high-precision acceleration sensors were used, which increased the complexity and cost of the system. To overcome the wind load disturbance on large radio telescopes, LQG and H∞ control methods [20,21] were designed, and they demonstrated better disturbance rejection performance than PID, however, at the expense of phase margin stability. In addition, their tuning for antenna tracking purposes is a tricky process.

The nonlinear disturbance observer (NDOB) method was first proposed to estimate the disturbance torque caused by unknown friction in nonlinear robotic manipulators [22]. Subsequently, an NDOB with an exponential rate of convergence was introduced by constructing a nonlinear observer gain function [23]. To overcome the limitations of linear DOBs in the presence of highly nonlinear and coupled dynamics, researchers have started to investigate NDOBs for systems with nonlinear dynamics during the past decade. For example, ref. [24] demonstrated the design and application of some nonlinear disturbance observers. To strengthen the disturbance rejection ability of flexible air-breathing hypersonic vehicles, a new NDOB was constructed through an NTD-based on the hyperbolic sine function to enhance the back-stepping controller’s robustness [25]. A new NDOB is exploited and, combining it with back-stepping, was utilized to achieve robust control of velocity and altitude [26]. They demonstrated that the NDOB constructed by different nonlinear methods achieves robustness. However, the NDOB in [25,26] has too many parameters to be adjusted, which is not conducive to use in engineering practice.

In this study, an NDOB with an exponential rate of convergence was developed which only required tuning for its gain parameter. On the basis of the classic three-closed-loop control structure of telescope servo system, a novel cascade anti-disturbance structure (NCADS) with an LADRC on the speed loop and a nonlinear disturbance observer (NDOB) on the current loop was proposed. Combining LADRC with NDOB solved the anti-disturbance problems under limited bandwidths. The inner current loop is much faster than the outer speed loop, so the dominant disturbance was firstly compensated for by the NDOB through feedforwarding it to the current reference without acceleration sensors, and then the residual disturbance was rejected by the LADRC of the speed loop, which lightened the burden of the LESO. The NCADS method achieves better anti-disturbance performance compared to the conventional proportional integral (PI) + NDOB and LADRC method.

The tracking differentiator (TD) is an important component of ADRC [8]. It has two main functions: one is to construct differentiators, and the other is to plan the transition process. Many previous studies on TDs mainly focus on constructing differentiators [27,28,29]; however, there are rarely studies about the use of TDs to plan transition processes. A variable parameter linear tracking differentiator (VLTD) with speed and acceleration bounds was applied in large ground-based telescopes in [30], which achieved almost the same performance as the NTD. In the study, an improved NTD with upper bounds on speed and acceleration was proposed to smoothen and speed up the position response and was shown to have better performance for planning transition processes than the NTD.

Simulations and experiments of the proposed control method demonstrated better performance on the main axis control of a 2.5-m telescope driven by PMSMs. The main contributions of this paper are summarized as follows:

(1) The proposed NCADS with an LADRC on the speed loop and an NDOB on the current loop enhances the anti-disturbance performance under limited bandwidth.
(2) The improved NTD with the upper bounds on speed and acceleration achieves a fast and smooth transition process for the position loop.
(3) The proposed method has few parameters and is simple to tune based on control bandwidth, which makes it easy for engineering applications.

The remainder of this paper is organized as follows: Section 2 highlights the dynamic models and controller design. Section 3 presents the simulation results and analysis. The experimental results and discussion are presented in Section 4. Finally, the conclusions of the study are provided in Section 5.

## 2. Servo Control System Design of a Large Ground-Based Telescope

**Figure 1:**
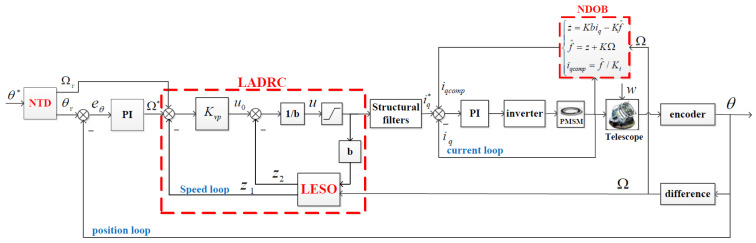
Block diagram of a ground-based telescope servo control system (note: * indicates the reference value).

Because the servo systems for the azimuth and elevation axes of a telescope are independent of each other, in this study, the elevation axis of a telescope was considered as an example. Figure 1 shows the diagram of a typical telescope servo control system, which is mainly composed of a position loop, a speed loop, a current loop, an inverter, a PMSM, and the telescope. The transition process of the position loop is based on the improved nonlinear tracking differentiator (NTD), which generates the reference position and velocity according to the maximum speed and acceleration allowed by the telescope. The smoothed position reference is used as the new command, and the speed reference is used to feedforward speed. The linear active disturbance rejection control (LADRC) is used on the speed loop, and the linear extended state observer (LESO) estimates and compensates the disturbance. Moreover, the conventional proportional integral (PI) control is used on the current loop. Although the use of acceleration information can improve the tracking performance of the telescope, a high-precision acceleration sensor is expensive and requires high installation accuracy. Therefore, to improve the servo performance without an acceleration sensor, an NDOB is designed to estimate and compensate for the disturbance. Since the NDOB compensates for most of the disturbance through feedforward q-axis current command, the LESO only needs to estimate the residual disturbance on the outer speed loop. The ADRC + NDOB method has a better anti-disturbance performance, and therefore the speed loop can achieve higher low-speed tracking precision under disturbances. Because the control bandwidth of the speed loop is limited by the resonant frequency of the telescope, the design of the structural filter is necessary, for which the reader is referred to [31], as it is not covered here.

### 2.1. Mathematical Model of Vector Control for PMSM

Ground-based, large-aperture telescope servo systems are mainly used in high-precision and low-speed applications, and a surface mount PMSM is generally used. The mathematical model of a PMSM in the d–q coordinate system is expressed as follows.

Voltage equation:

(1) $$\left\{\begin{array}{c} u_{q}=R_{s}i_{q}+L_{q}\frac{di_{q}}{dt}+\omega_{e}\left(L_{d}i_{d}+\psi_{f}\right),\\ u_{d}=R_{s}i_{d}+L_{d}\frac{di_{d}}{dt}+\omega_{e}L_{q}i_{q} \end{array}\right.$$

Electromagnetic torque equation:

(2) $$T_{e}=\frac{3}{2}n_{p}\psi_{f}i_{q}=K_{t}i_{q},$$

Motion equation:

(3) $$J\frac{d\Omega }{dt}=T_{e}-T_{L}-B\Omega ,$$

where $u_{d}$ and $u_{q}$ are the voltages; $i_{d}$ and $i_{q}$ are the currents; and $L_{d}$ and $L_{q}$ are the inductance along the *d* and *q* axes, respectively; furthermore, $L_{q}=L_{d}=L$. $R_{s}$ is the phase resistance of the stator; $\psi_{f}$ is the permanent magnet flux; $n_{p}$ is the number of pole pairs; $K_{t}$ is the torque coefficient; $\omega_{e}$ is the electrical angular speed of rotor rotation; Ω is the mechanical angular speed of rotor rotation; and $\omega_{e}=n_{p}\Omega$. $T_{L}$ is the load torque, and *B* is the coefficient of viscous friction.

### 2.2. Design of PI Controller of Current Loop

The AC servo system of the 2.5-m-aperture telescope has a low-speed application and usually adopts the cascade control of the position, speed, and current. The inner current loop has a higher bandwidth than the outer loops of speed and position, and the PI control can meet the requirements for current response. The vector control method is adopted, and the influence of the back electromotive force is ignored. The open-loop transfer function for the current loop is given as follows:

(4) $$G_{co}\left(s\right)=K_{cp}\left(1+\frac{1}{\tau_{i}s}\right)\left(\frac{1}{L_{q}s+R_{s}}\right)$$

According to the type-I system settings, $\tau_{i}=\tau_{e}=L_{q}/R_{s}$, the closed-loop transfer function of the current loop is given as

(5) $$G_{cc}\left(s\right)=\frac{G_{co}\left(s\right)}{1+G_{co}\left(s\right)}=\left(\frac{1}{(L_{q}/K_{cp})s+1}\right).$$

The appropriate bandwidth $\omega_{cc}=K_{cp}/L_{q}$ of the current loop can be designed by adjusting proportional gain $K_{cp}$. For a 2.5-m telescope, the closed-loop bandwidth of the current loop is about 100 Hz.

After the current loop is set, ignoring the influence of the disturbance torque, the transfer function of the controlled object on the speed loop is given as follows:

(6) $$G_{vplant}\left(s\right)=\frac{\Omega (s)}{I_{q}\left(s\right)}=\frac{K_{t}}{\left(Js+B\right)\left(\tau_{c}s+1\right)}.$$

For large-aperture telescopes, the current loop has a much higher bandwidth than the speed loop. By ignoring its time constant, the speed loop can be further simplified into a first-order system, as follows:

(7) $$\dot{\Omega }=\frac{K_{t}}{J}i_{q}-\frac{B}{J}\Omega .$$

The current is used as the system input *u*, the mechanical angular speed is used as the system output Ω, and a random disturbance *w* is introduced. The system parameter is denoted by $a_{0}=B/J$, and the system control gain is denoted by $b_{0}=K_{t}/J$. Thus, the speed loop model becomes

(8) $$\dot{\Omega }=b_{0}u-a_{0}\Omega +w.$$

Considering the control gain error, the controller gain b is introduced to obtain

(9) $$\dot{\Omega }=\left(b_{0}-b\right)u-a_{0}\Omega +w+bu.$$

### 2.3. Design of the NDOB for Feedforward Compensation on the Current Loop

The total system disturbance is denoted as $f=\left(b_{0}-b\right)u-a_{0}\Omega +w$. The torque disturbance of the servo system for the telescope includes friction, cogging torque, wind load, and random disturbance. The total disturbance includes the internal and external disturbances of the system, including the known and unknown parts. The system model can be expressed as follows:

(10) $$\dot{\Omega }=f+bu,$$

Because the total disturbance cannot be measured directly, it must be estimated by the disturbance observer (DOB) as follows:

(11) $$\begin{array}{c} \dot{\hat{f}}=K\left(f-\hat{f}\right)=-K\hat{f}+Kf\\ =-K\hat{f}+K\left(\dot{\Omega }-bu\right), \end{array}$$

Generally, there is no prior knowledge of the differential of the disturbance, which changes slowly with respect to the dynamic characteristics of the DOB, assuming $\dot{f}=0$. The error of the DOB is given by

(12) $$\begin{array}{c} \tilde{f}=\left(f-\hat{f}\right). \end{array}$$

Thus, we obtain

(13) $$\begin{array}{c} \dot{\tilde{f}}=-\dot{\hat{f}}=-K\left(f-\hat{f}\right)=-K\tilde{f} \end{array}$$

The DOB error satisfies the following constraints.

(14) $$\begin{array}{c} \dot{\tilde{f}}+K\tilde{f}=0 \end{array}$$

The DOB converges exponentially, and the convergence rate can be determined by the gain K. In practical engineering, it is difficult to obtain the acceleration signal $\dot{\Omega }$ by differentiating the speed because of the signal noise. Therefore, the DOB cannot estimate disturbance well, but the NDOB can be designed without the acceleration information.

The auxiliary parameter of the NDOB is defined as

(15) $$\begin{array}{c} z=\hat{f}-K\Omega . \end{array}$$

Differentiating both sides of Equation (15) and combining with Equation (11), we have

(16) $$\begin{array}{c} \dot{z}=\dot{\hat{f}}-K\dot{\Omega }=K\left(\dot{\Omega }-bu\right)-K\hat{f}-K\dot{\Omega }=-Kbu-K\hat{f}. \end{array}$$

The NDOB is designed as:

(17) $$\left\{\begin{array}{c} \dot{z}=-Kbu-K\hat{f}\\ \hat{f}=z+K\Omega \end{array}\right.$$

The convergence of NDOB is proven below:

(18) $$\begin{array}{c} \dot{\tilde{f}}=-\dot{\hat{f}}=-\dot{z}-K\dot{\Omega }=Kbu+K\hat{f}-K\dot{\Omega }\\ =K\left(\hat{f}-f\right)=-K\tilde{f} \end{array}$$

We also obtain Equation (14) and solve it as follows:

(19) $$\begin{array}{c} \tilde{f}\left(t\right)=\tilde{f}\left(t_{0}\right)e^{-Kt}. \end{array}$$

Since the value of $\tilde{f}\left(t_{0}\right)$ is bounded, $\tilde{f}\left(t\right)$ will converge exponentially, and the convergence rate can be determined by gain *K*. A larger value of *K* provides a larger range of estimated disturbance frequencies but results in a control system that is more sensitive to noise. Thus, the disturbance *f* can be estimated by Equation (17) of the NDOB without acceleration information.

### 2.4. Design of the LADRC for the Speed Loop

After the disturbance is compensated for by the NDOB, the disturbance rejection performance is improved, and only the residual error $\tilde{f}\left(t\right)$ needs to be estimated instead of the total disturbance *f*. This reduces the burden the ADRC takes on while estimating the disturbance. The system model can be expressed as follows:

(20) $$\dot{\Omega }=\tilde{f}+bu,$$

Design a second-order LESO for the first-order system:

(21) $$\left\{\begin{array}{c} e_{\Omega }=\Omega -z_{1}\\ \dot{z_{1}}=z_{2}+\beta_{1}e_{\Omega }+bu\\ \dot{z_{2}}=\beta_{2}e_{\Omega } \end{array}\right.$$

where $z_{1}$ is the estimated value of the mechanical angular speed signal Ω, $e_{\Omega }$ is the estimation error of the LESO of the speed, $z_{2}$ is the estimated value of the disturbance $\tilde{f}$, and *u* is the system control output, which is a finite value in an actual physical system and its amplitude should therefore be limited. $\beta_{1}$ and $\beta_{2}$ are the LESO gains, which are determined by the angular frequency bandwidth $\omega_{vo}$ of the LESO. Thus, the eigenvalue of the LESO characteristic equation is $-\omega_{vo}$, which is expressed as follows:

(22) $$\left\{\begin{array}{c} \beta_{1}=2\omega_{vo}\\ \beta_{2}=\omega_{vo}^{2} \end{array}\right.$$

The error feedback control law is designed as a proportional controller:

(23) $$u_{0}=K_{vp}\left(\Omega^{*}-z_{1}\right),$$

where $\Omega^{*}$ is the reference input of mechanical angular speed, and $K_{vp}$ is the proportional gain of the speed loop. The actual control output is obtained by disturbance feedforward compensation:

(24) $$u=(u_{0}-z_{2})/b$$

The closed-loop transfer function of the speed loop can be obtained by substituting Equations (23) and (24) into Equation (21) by selecting the appropriate values of $\beta_{1}$ and $\beta_{2}$:

(25) $$G_{vc}\left(s\right)=\frac{\Omega (s)}{\Omega^{*}\left(s\right)}=\frac{1}{(1/K_{vp})s+1},$$

where $K_{vp}=\omega_{vc}$. The controller gain $K_{vp}$ is determined according to the required angular frequency bandwidth $\omega_{vc}$ of the speed loop. In general, $\omega_{vc}$ is set to one-third of the mechanical anti-resonance frequency.

### 2.5. Design of the PI Controller for the Position Loop

The diagram of the position loop control is shown in Figure 1. After the speed loop is set, the transfer function of the controlled object on the position loop is expressed as follows:

(26) $$G_{pplant}\left(s\right)=\frac{\theta (s)}{\Omega^{*}\left(s\right)}=\frac{1}{s(\left(1/K_{vp}\right)s+1)}.$$

The position loop controller is designed as a PI controller. Considering the small integration gain, to simplify the analysis, the integral term is ignored. Thus, the closed-loop transfer function of the position loop becomes

(27) $$G_{pc}\left(s\right)=\frac{\theta (s)}{\theta_{r}\left(s\right)}=\frac{K_{vp}K_{pp}}{s^{2}+K_{vp}s+K_{vp}K_{pp}}.$$

If the proportional gain of the position loop is selected $K_{pp}=\frac{\omega_{vc}}{4}$, the transfer function of position loop is

(28) $$G_{pc}\left(s\right)=\frac{\theta (s)}{\theta_{r}\left(s\right)}=\frac{{\left(\frac{\omega_{vc}}{2}\right)}^{2}}{{\left(s+\frac{\omega_{vc}}{2}\right)}^{2}}.$$

The closed-loop angular frequency bandwidth of the position loop will be set as follows:

(29) $$\omega_{pc}=0.322\omega_{vc}.$$

### 2.6. Design Transition Process Using the NTD

The position loop uses the NTD to generate the transition process, and the reference position and speed are planned according to the maximum acceleration and maximum speed to suppress resonance and reduce overshoot. Here, the NTD adopts Han’s discrete fastest control function fhan [8]. The discrete fastest tracking function $fhan(x_{1},x_{2},r,h)$ is given by

(30) $$\left\{\begin{array}{c} d=rh\\ d_{0}=hd\\ y=x_{1}+hx_{2}\;\\ a0=\sqrt{d^{2}+8r\left|y\right|}\;\\ a=\left\{\begin{array}{c} x_{2}+\frac{\left(a_{0}-d\right)}{2}sign\left(y\right),\left|y\right|>d_{0}\;\\ x_{2}+\frac{y}{h},\left|y\right|\leq d_{0}\; \end{array}\right.\\ fhan=\left\{\begin{array}{c} rsign(a),|a|>d\;\\ r\frac{a}{d},\left|a\right|\leq d \end{array}\right. \end{array}\right.$$

The NTD is improved according to the maximum speed and maximum acceleration limit of the telescope, and the design is as follows:

(31) $$\left\{\begin{array}{c} fh=fhan(x_{1}\left(k\right)-v\left(k\right),x_{2}\left(k\right),r,h_{0})\\ x_{1}\left(k+1\right)=x_{1}\left(k\right)+hx_{2}\left(k\right)\\ x_{2}\left(k+1\right)=x_{2}\left(k\right)+hfh\\ x_{2}\left(k+1\right)=sat\left(x_{2}\left(k+1\right)\right)\\ sat\left(x_{2}\right)=sign\left(x_{2}\right)min\left\{x_{2max},\left|x_{2}\right|\right\} \end{array}\right.$$

where *v* is the input signal to be followed and *r* is the speed factor. For a larger value of *r*, the $x_{1}$ value reaches the set value *v* faster. The turning frequency $\omega_{TD}$ of the NTD is also determined by *r*. Generally, $\omega_{TD}=1.14\sqrt{r}$, and *r* is taken as the maximum acceleration of the telescope, that is, $r=a_{max}$. $x_{2}$ can be used as the approximate derivative of *v*. *h* is the integration step, which is generally the sampling period. $h_{0}$ is the filtering factor. Generally, $h_{0}=mh$, and *m* is a positive integer greater than 1, which can filter out the noise of the input signal. If a larger *m* is selected, the filtering performance is better, but the phase lag is higher. sat() is the amplitude limiting function, and the position signal $\theta^{*}$ is the signal to be followed, where $x_{2max}$ is the maximum allowable mechanical angular speed $\Omega_{max}$ of the system.

## 3. Simulation and Analysis

In this section, we present the simulation results of the elevation axis servo system of a 2.5-m-wide field survey telescope. The servo system parameters are given in Table 1.

The PI parameters of the current loop are as follows:

$$K_{cpq}=K_{cpd}=\omega_{cc}L_{q}=14.9,\tau_{iq}=\tau_{id}=L_{q}/R_{s}=0.009694.$$

After the current loop is set, the transfer function of the speed loop controlled object is obtained as

(32) $$G_{vplant}\left(s\right)=\frac{118}{\left(7100\;s+30)(0.0016\;s+1\right)}.$$

**Figure 2:**
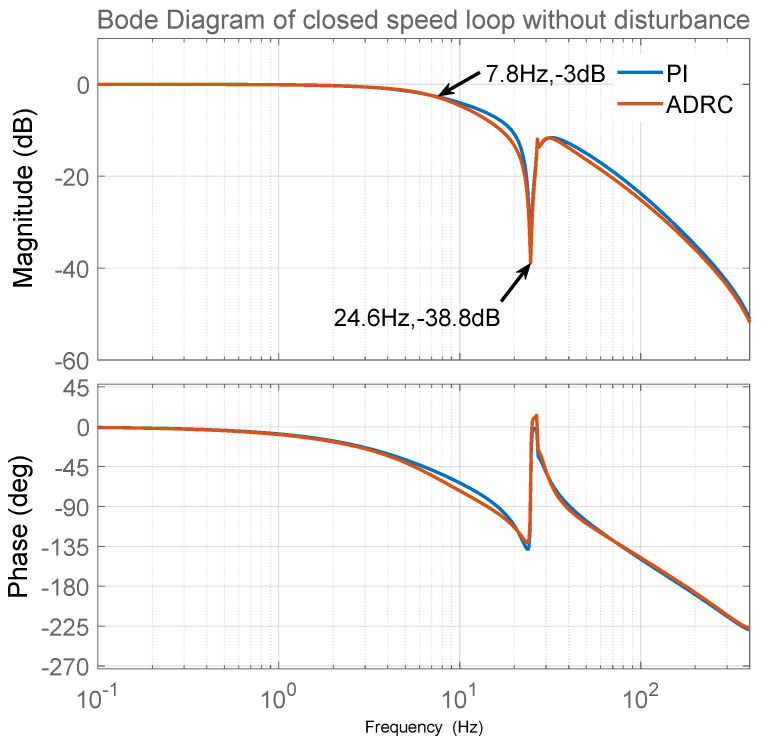
Bode diagram of speed loop without disturbance.

For fair comparison, PI has the same closed-loop bandwidth of the speed loop as that of the LADRC. As shown in Figure 2, in the absence of disturbance, the closed-loop bandwidth of the speed loop is set to 7.8 Hz when the mechanical anti-resonance frequency is 24.6 Hz. The parameters of the PI control are as follows:

$$K_{vp-PI}=1324,K_{vi-PI}=8.0,$$

The parameters of LADRC are as follows:

$$b=\frac{K_{t}}{J}=0.01662,K_{vp-LADRC}=\omega_{vc}=40.0,\omega_{vo}=\omega_{vc}=40.0$$

### 3.1. Main Disturbance of the Servo Systems of Large Telescopes

In telescope servo systems, the internal disturbance mainly includes nonlinear friction and cogging torque, whereas the external disturbance mainly includes wind load disturbance. When friction disturbance is considered in the 2.5-m telescope servo system, the friction torque can be expressed by the LuGre static model in Equation (33), and its parameters are shown in Table 2.

(33) $$T_{friction}\left(t\right)=F_{c}+\left(F_{s}-F_{c}\right)e^{-{\left(\Omega /v_{s}\right)}^{2}}+B\Omega .$$

Cogging torque is a kind of pulsating torque, basically generated by the interaction of the rotor’s magnetic flux and angular variations in the stator magnetic reluctance. The cogging torque of the PMSM can be expressed as follows:

(34) $$T_{cogging}\left(t\right)=\sum_{i=1}^{n}T_{cogi}sin\left(iN_{c}\theta \right).$$

where $N_{c}$ is the least common multiple between the number of slots and pole pairs, which is $N_{c}=270$ here, and $T_{cogi}$ is the amplitude of the ith-order harmonic cogging torque. The cogging torque only has an AC component and no DC component.

Considering the windward area of different elevation axis structures, we estimated the wind load at about 350 N·m when the wind speed is 15 m/s. The energy of wind is mainly concentrated in the frequency below 1 Hz. Therefore, the time-domain expression of wind disturbance is constructed by means of average wind (${\bar{T}}_{wind}$ = 350 N·m) plus random wind, which is generated by white noise filtered by a low-pass filter with a bandwidth of 1 Hz, and the random wind $T_{stochastic}\left(t\right)=\pm 15$ N·m.

(35) $$T_{wind}\left(t\right)={\bar{T}}_{wind}+T_{stochastic}\left(t\right).$$

### 3.2. Simulation of Disturbance Estimation and Rejection Performance

In the case where there is no acceleration sensor, the NDOB is designed to estimate the dominant disturbance through first feedforward compensating the *q* axis reference current, and then the LADRC estimates the residual disturbance on the speed loop. The NDOB’s parameters are as follows:

$$K=62.8,b=\frac{K_{t}}{J}=0.016620.$$

**Figure 3:**
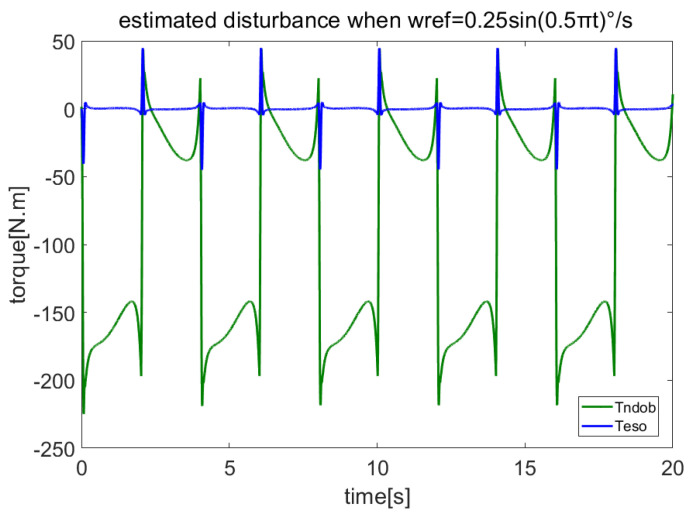
Disturbance estimated by NDOB and LADRC when tracking sine speed.

**Figure 4:**
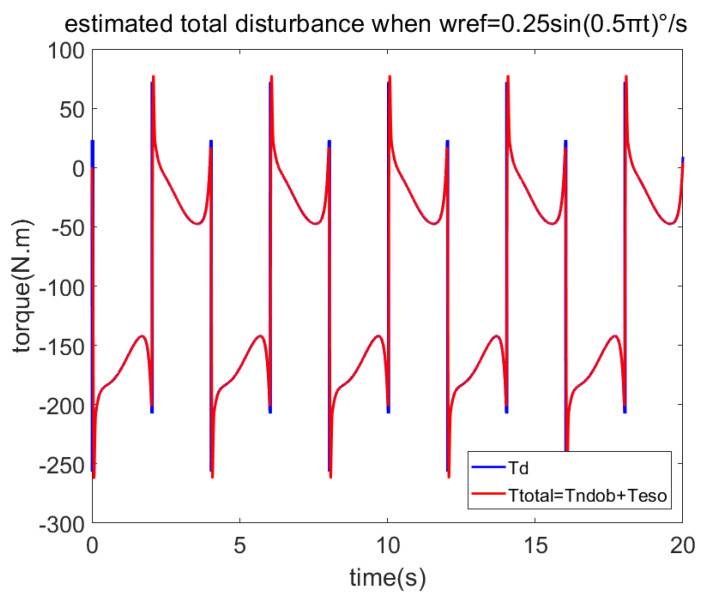
Total disturbance estimated by NDOB + LADRC when tracking sine speed.

**Figure 5:**
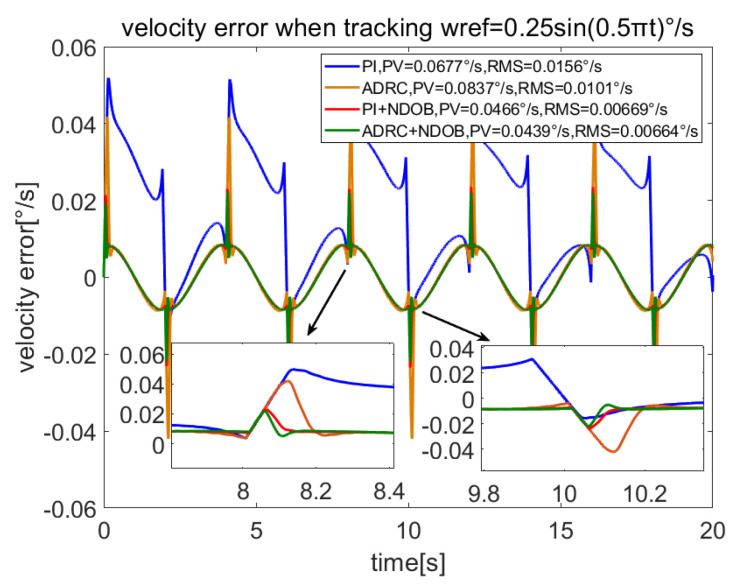
Comparison of velocity errors of different methods when $\omega_{ref}=0.25sin\left(0.5\pi t\right){}^{\circ}/s$.

In order to verify the anti-disturbance ability of the proposed method on friction and cogging torque, let the servo system track the sine speed curve $w_{ref}=0.25sin(0.5$πt). The estimated disturbances by NDOB and ESO are shown in Figure 3, while the total load and the estimated total disturbance are shown in Figure 4. Figure 3 shows that the NDOB estimated the dominant disturbance, while ESO only estimated the residual disturbance. Moreover, Figure 4 shows that NDOB + ADRC can estimate the total disturbance well. Figure 5 compares the velocity errors of different control methods. It shows that the RMS velocity error of PI is largest, and its average value is not zero. Due to the ADRC having certain anti-disturbance ability, its error is smaller. Because PI + NDOB has further improved anti-disturbance, its error is further reduced. ADRC + NDOB has the best anti-disturbance performance of the four control methods, and the corresponding error PV value and RMS value are also the smallest.

**Figure 6:**
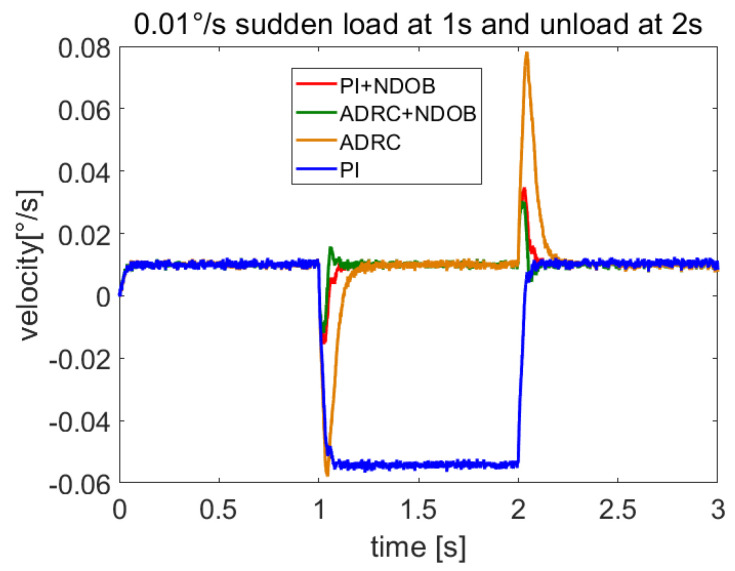
Response at 0.01°/s speed to sudden wind loading and unloading.

**Figure 7:**
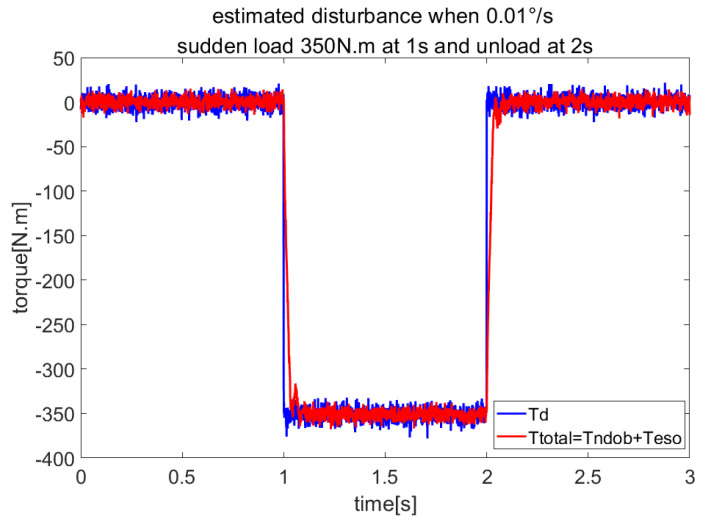
Estimated load by NDOB + ADRC under sudden wind loading and unloading.

In order to verify the rejection of wind load disturbance, we let the servo system run at 0.01°/s and add sudden wind load using Equation (35) at 1 s and unloading at 2 s. The speed response is shown in Figure 6. The wind load and estimated total disturbance are shown in Figure 7. The comparison of anti-disturbance performance under sudden wind load is shown in Table 3. The results show that NDOB + ADRC has smaller speed fluctuations and a shorter adjustment time than the other three methods, and it is clear that NDOB + ADRC has a better disturbance-rejection performance.

### 3.3. Simulation of Transition Process Planed by the Improved NTD

The parameters of the transition process are as follows: maximum acceleration, $a_{max}=7{}^{\circ}/s^{2}$; maximum speed, $\Omega_{max}=10{}^{\circ}/s$; and the sampling frequency of speed loop is 1000 Hz. Therefore, the integral step is taken as h=0.001. The improved NTD function is chosen as $fhan(\theta_{r}-\theta^{*},\Omega_{r},a_{max},2h)$.

**Figure 8:**
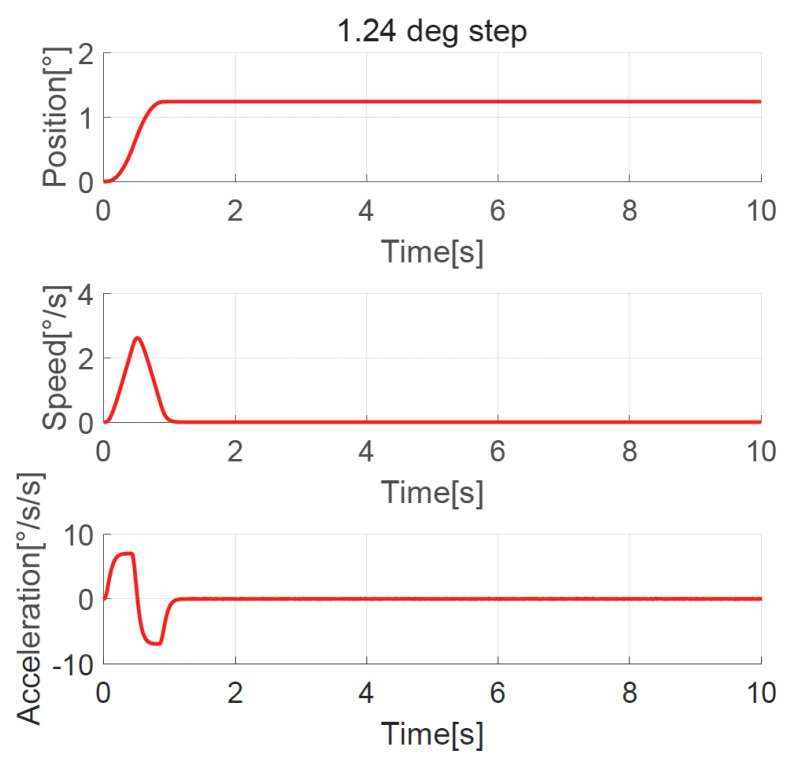
Position, speed, and acceleration curve using the 1.24° FOV step response with improved NTD.

**Figure 9:**
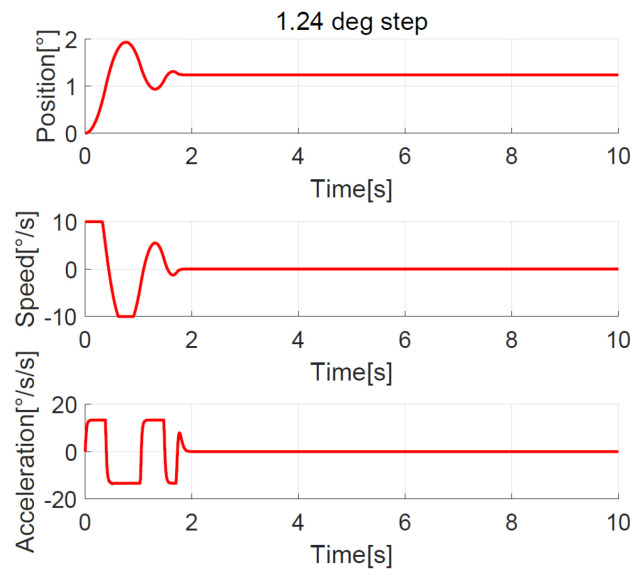
Position, speed, and acceleration curve using the 1.24° FOV step response without NTD.

**Figure 10:**
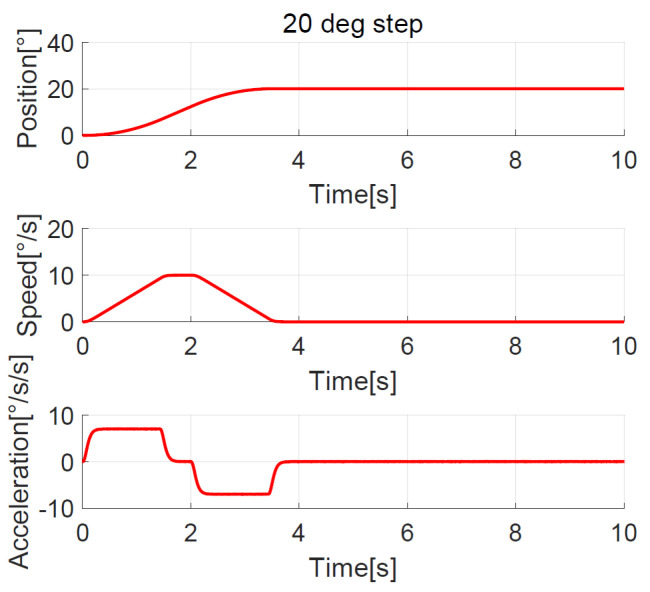
Position, speed, and acceleration curve using the 20° step response with improved NTD.

**Figure 11:**
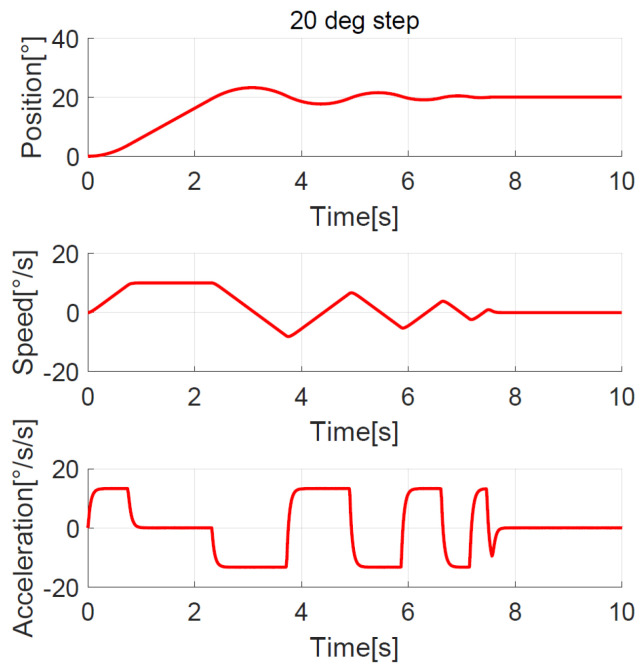
Position, speed, and acceleration curve using the 20° step response without NTD.

The position, speed, and acceleration response curves of the 1.24° field of view (FOV) step are shown in Figure 8. The planned speed curve of the improved NTD is triangular. The system accelerates at the maximum acceleration $a_{max}=7{}^{\circ}/s^{2}$ first and slows down before the speed reaches the maximum, and the adjustment time is about 1.0 s. The position, speed, and acceleration response curves of the 1.24° FOV step when the improved NTD is not used to plan the transition process are shown in Figure 9. There are two large jumps in the speed, and the adjustment time is about 2.0 s. The position, speed, and acceleration response curves of the 20° step are shown in Figure 10. The planned speed curve of the improved NTD is trapezoidal. The system accelerates at the maximum acceleration $a_{max}=7{}^{\circ}/s^{2}$, runs at a constant speed when the speed reaches the maximum $\Omega_{max}=10{}^{\circ}/s$, and then slows down. The adjustment time is about 4.0 s. The position, speed, and acceleration response curves of the 20° step when the improved NTD is not used to plan the transition process are shown in Figure 11. There are many large jumps in the speed, and the adjustment time is about 8.0 s. It can be observed that, when the improved NTD is used to plan the transition process, the speed response is smoother, and the adjustment time is shorter for both the small and large position steps.

## 4. Experimental Results and Discussions

### 4.1. Experiment Setup

**Figure 12:**
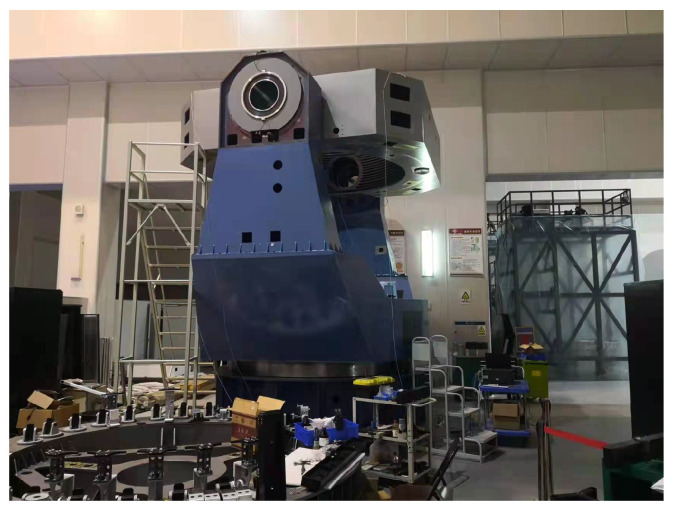
The ground-based 2.5-m-wide field survey telescope.

**Figure 13:**
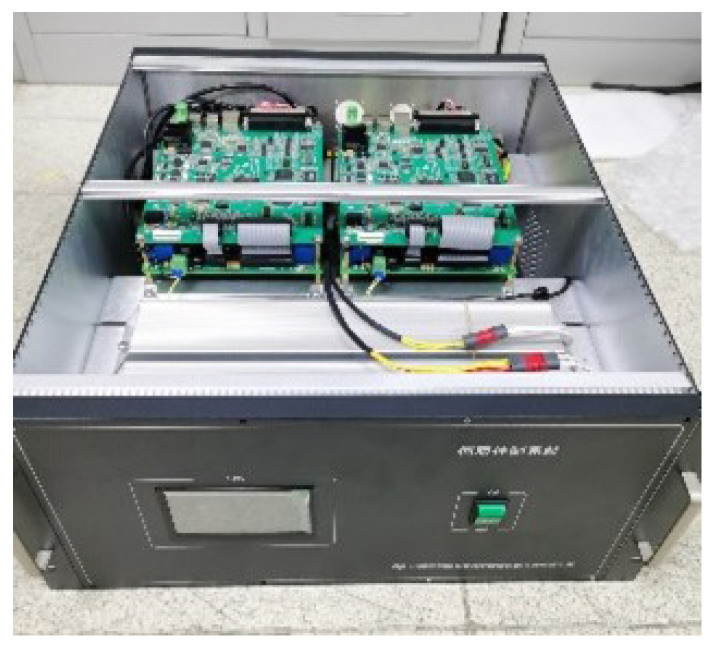
The servo controller and driver of 2.5-m-wide field survey telescope.

**Figure 14:**
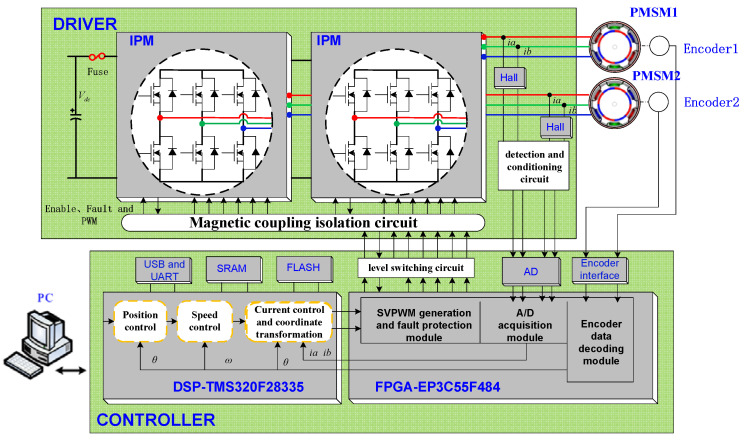
Configuration of the DSP and FPGA-based experimental circuit.

To verify the effectiveness of the proposed method, experiments were performed on a 2.5-m-wide field survey telescope. The telescope and its servo controller and driver are shown in Figure 12 and Figure 13, respectively. The elevation axis of the telescope is directly driven by PMSMs. A 32-bit absolute photoelectric encoder is used as the position feedback device, and the speed signal is obtained by position signal difference and low-pass filtering. The controller adopts a DSP + FPGA architecture design, and the configuration of the experimental circuit is shown in Figure 14. The DSP serves as the main controller to complete the position loop, speed loop, and current loop correction, and the FPGA serves as the co-controller to complete fault protection and pulse width modulation. The driver adopts intelligent power modules. The maximum driving voltage is 400 V, and the maximum driving current is 50 A. The servo system uses a DC switching power supply, and the DC bus voltage is 60 V. The parameters of the servo system are shown in Table 1. The vector control electronic commutation is implemented based on the seven-section SVPWM, and the sampling frequency of the current loop is 10 kHz.

All the parameters of the current loop PI controller, speed loop, NDOB, and NTD are roughly the same as those in the simulation and thus are not described here. The parameters of the position loop PI controller are as follows:

$$K_{pp}=10.0,K_{pi}=0.0005$$

### 4.2. Anti-Disturbance Performance

**Figure 15:**
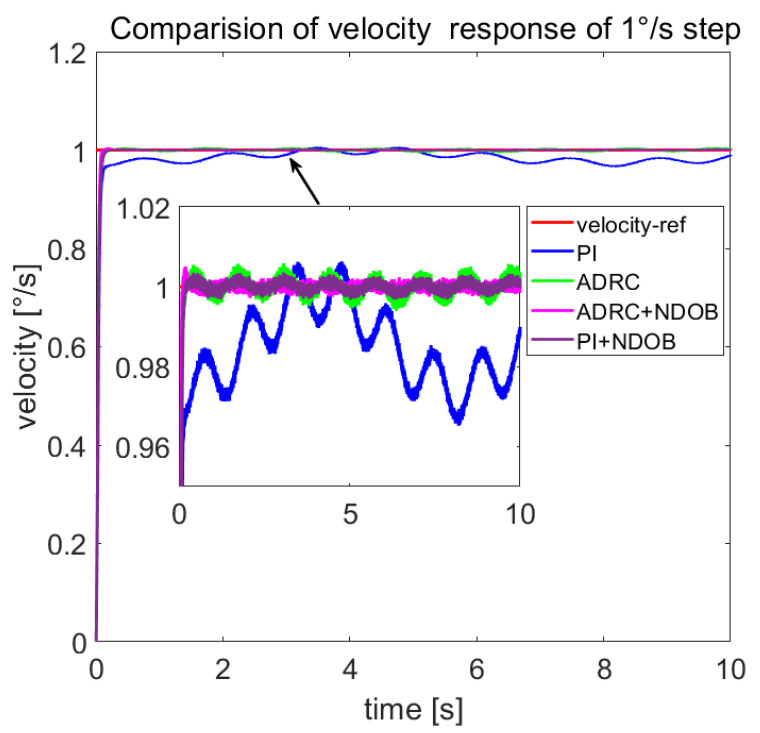
Comparison of 1°/s step responses under friction and cogging torque.

**Figure 16:**
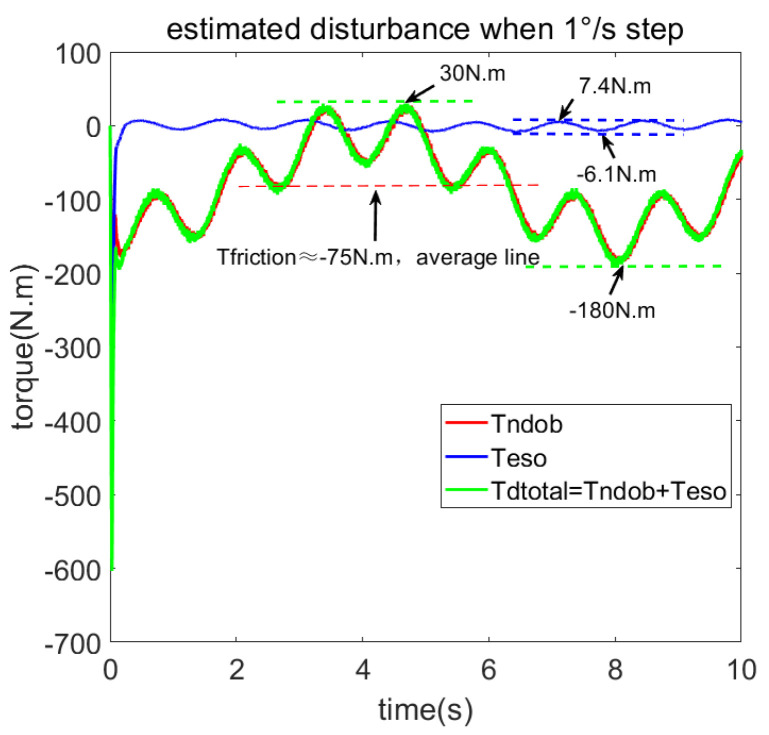
Load torque estimated by NDOB and ADRC when 1°/s.

**Figure 17:**
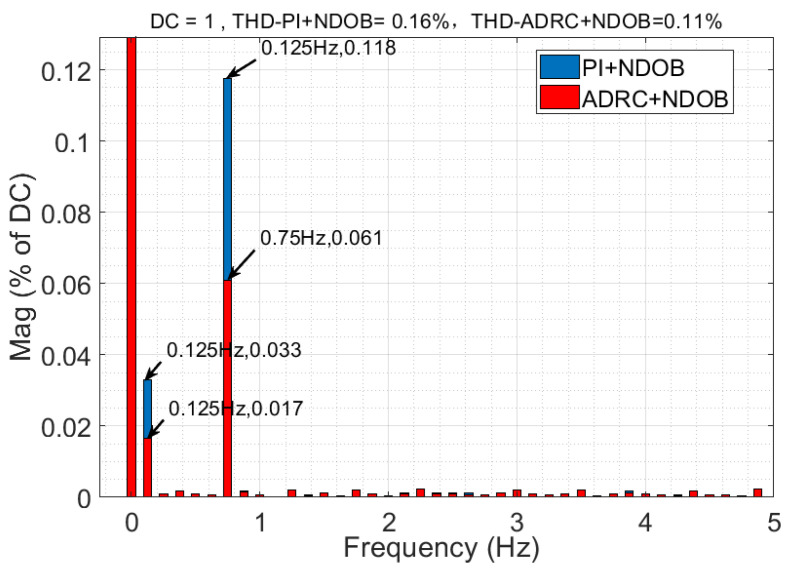
Comparison of speed harmonics analyzed by FFT.

The comparison of the 1°/s speed step response of the four control methods is shown in Figure 15, and the disturbance load estimated by the NDOB and ESO is shown in Figure 16. Figure 16 shows that the total disturbance estimated by NDOB + ADRC and the NDOB estimates the dominant disturbance, which ranges from −180 N·m to 30 N·m, while the ESO estimates the residual disturbance, which ranges from −6.1 N·m to 7.4 N·m. It can be observed that the average value of the estimated load is −75 N·m, which should be the friction torque, deducing that the amplitude of the cogging torque is about 105 N·m. As shown in Figure 14, the four control methods have approximate rise times but different steady-state responses. The PI method has the largest speed fluctuation, and the ADRC + NDOB method has the smallest speed ripples, thus exhibiting the best disturbance rejection of friction and cogging torque. The speed harmonics analyzed by FFT are shown in Figure 17. When the tracking speed is 1°/s, the frequency component corresponding to the number of pole pairs is 0.125 Hz, while the frequency component corresponding to the number of stator slots is 0.75 Hz, which matches Figure 17. Compared with PI + NDOB, the speed harmonics component of 0.125 Hz and 0.75 Hz of ADRC + NDOB was reduced by about 48%. At present, the 2.5-m telescope is still in the laboratory, so it has not been tested under wind disturbance.

### 4.3. Tracking Performance

#### 4.3.1. Position Response with Sine Guide to Verify Fast Tracking

**Figure 18:**
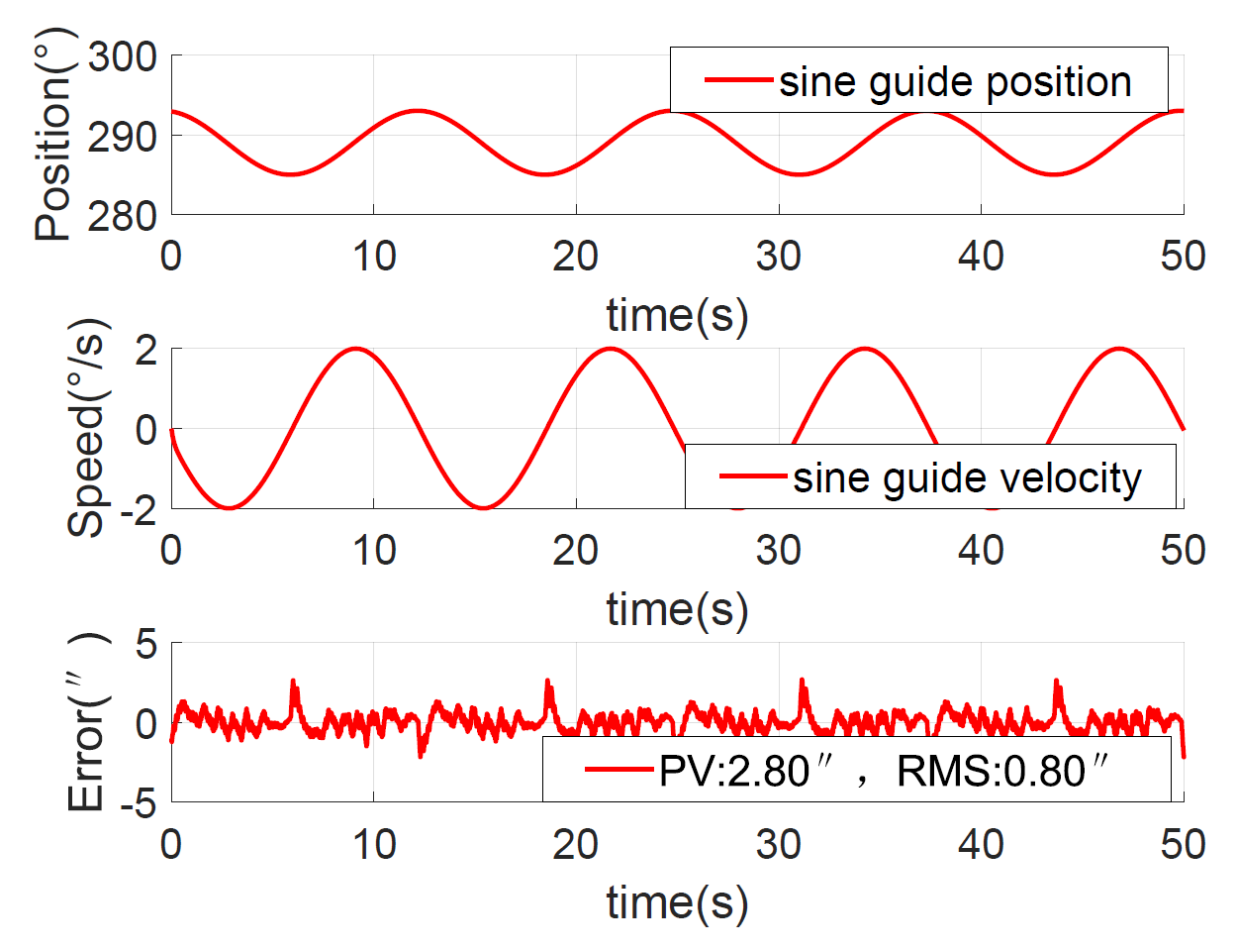
Position response for the sine command with ADRC + NDOB.

**Figure 19:**
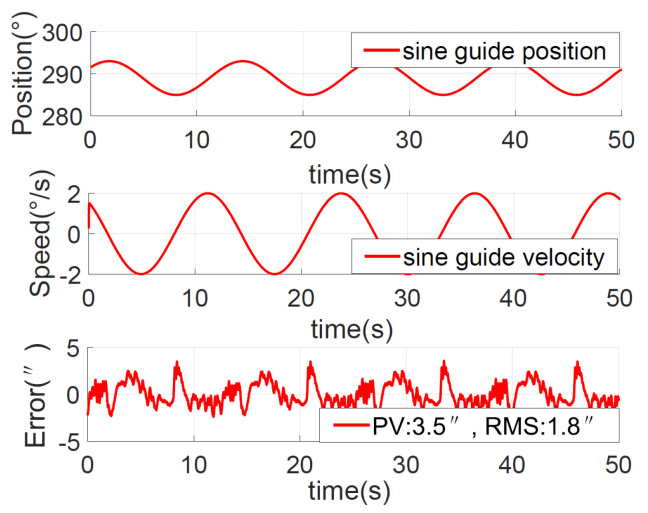
Position response for the sine command with PI + NDOB.

To verify the fast tracking performance, equivalent sinusoidal guidance was performed with a velocity of 2°/s and an acceleration of $1{}^{\circ}/s^{2}$, that is, $\theta^{*}=4{}^{\circ}sin\left(0.5t\right)$. The position, speed, and error curves of the ADRC + NDOB and PI + NDOB methods are shown in Figure 18 and Figure 19, respectively.

The equivalent sinusoidal guidance position error peak value of ADRC + NDOB is $2.80^{\prime \prime }$, and the RMS value is about $0.80^{\prime \prime }$. The PI + NDOB equivalent sinusoidal guidance position error peak value is $3.5^{\prime \prime }$ and the RMS value is about $1.8^{\prime \prime }$. The equivalent sinusoidal tracking error of ADRC + NDOB is smaller, and the tracking accuracy is higher than that of PI + NDOB.

#### 4.3.2. Position Response with Slope Guide to Verify Ultra-Low-Speed Tracking

**Figure 20:**
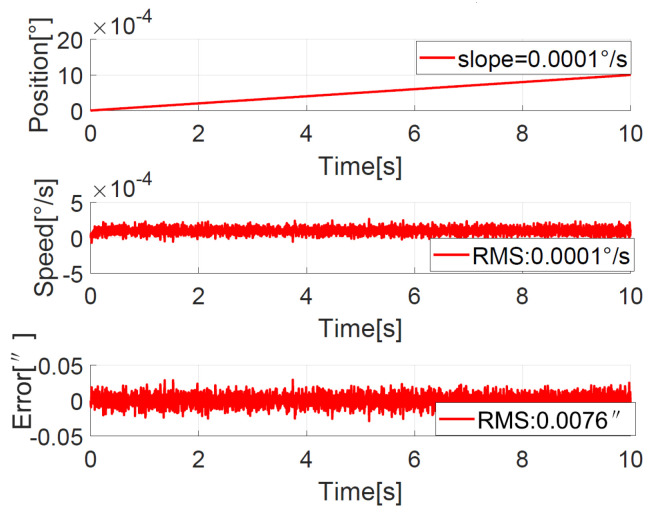
Position response for the slope command with ADRC + NDOB.

**Figure 21:**
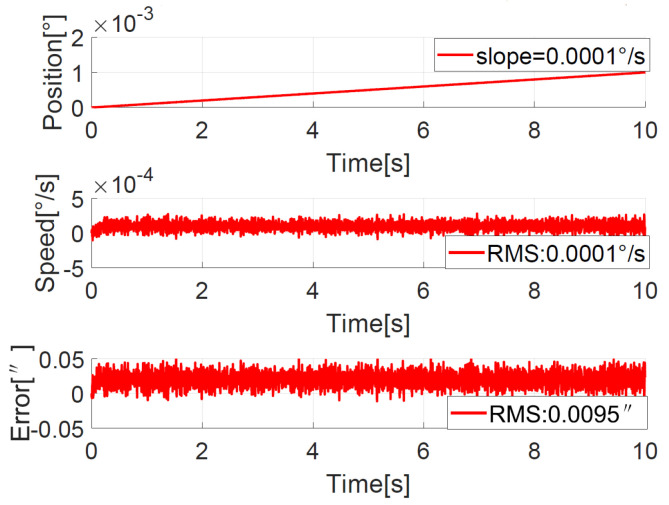
Position response for the slope command with PI + NDOB.

To verify the ultra-low-speed tracking performance, a position curve with a slope of 0.0001°/s is used as a guide, that is, $\theta^{*}=0.0001{}^{\circ}t$. The position, speed, and error curves of the ADRC + NDOB and PI + NDOB are shown in Figure 20 and Figure 21, respectively. As can be seen from the figures, the error RMS value of ADRC + NDOB is $0.0076^{\prime \prime }$, and the error RMS value of PI + NDOB is $0.0095^{\prime \prime }$. It can be seen that ADRC + NDOB has smaller ultra-low-speed tracking error and higher tracking accuracy than PI + NDOB.

### 4.4. Fast and Smooth Transition Process

**Figure 22:**
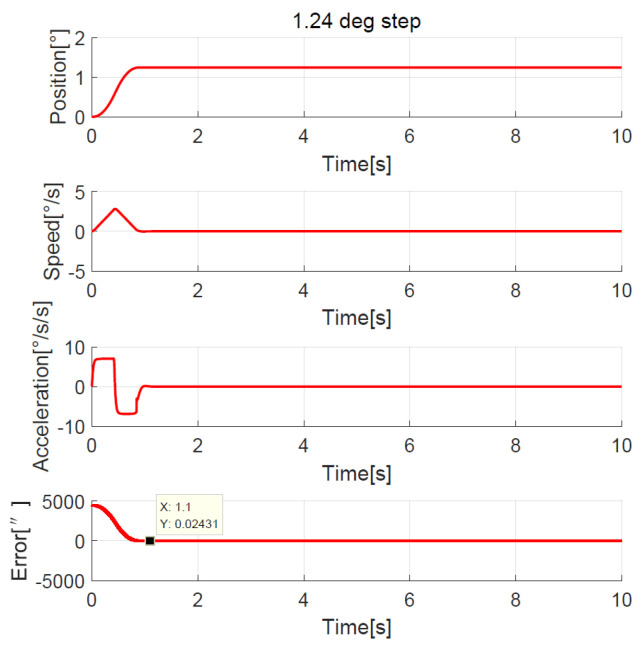
Experimental 1.24° FOV step response with improved NTD.

**Figure 23:**
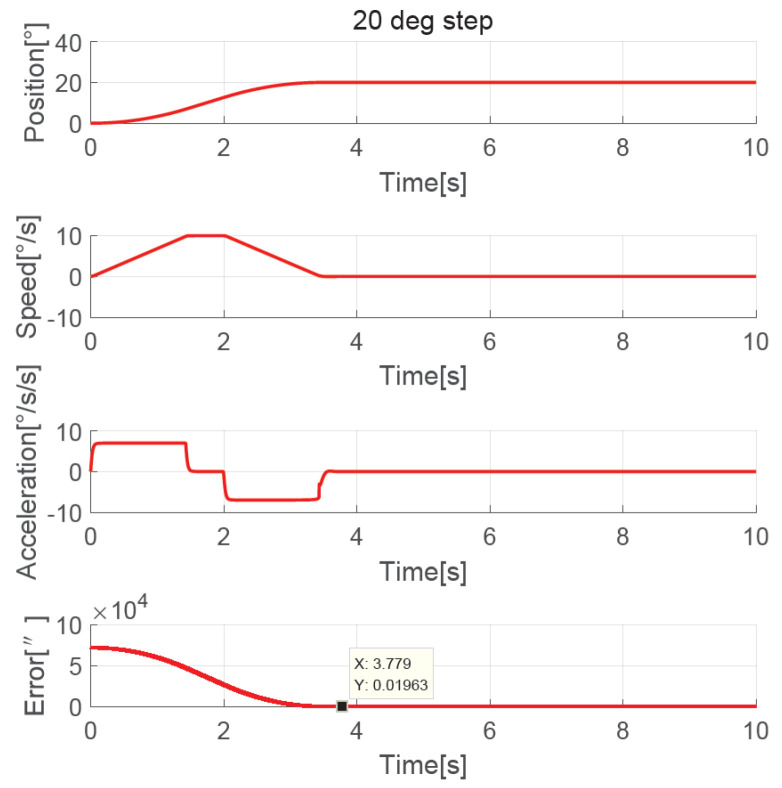
Experimental 20° step response with improved NTD.

**Figure 24:**
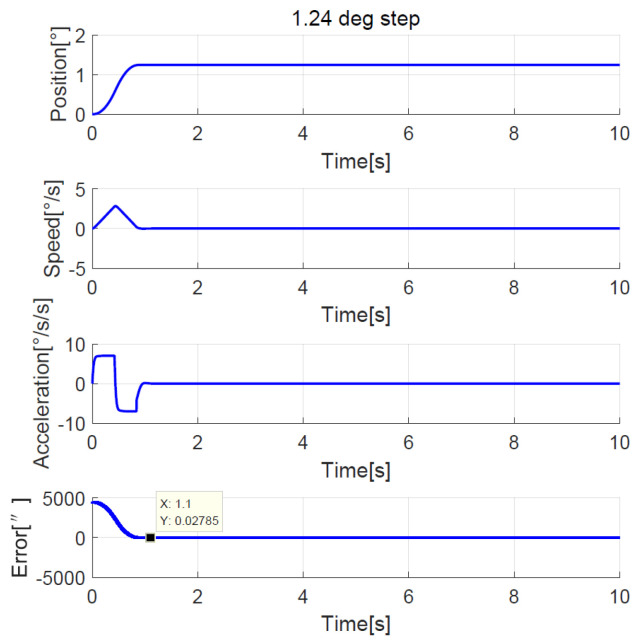
Experimental 1.24° FOV step response with conventional NTD.

**Figure 25:**
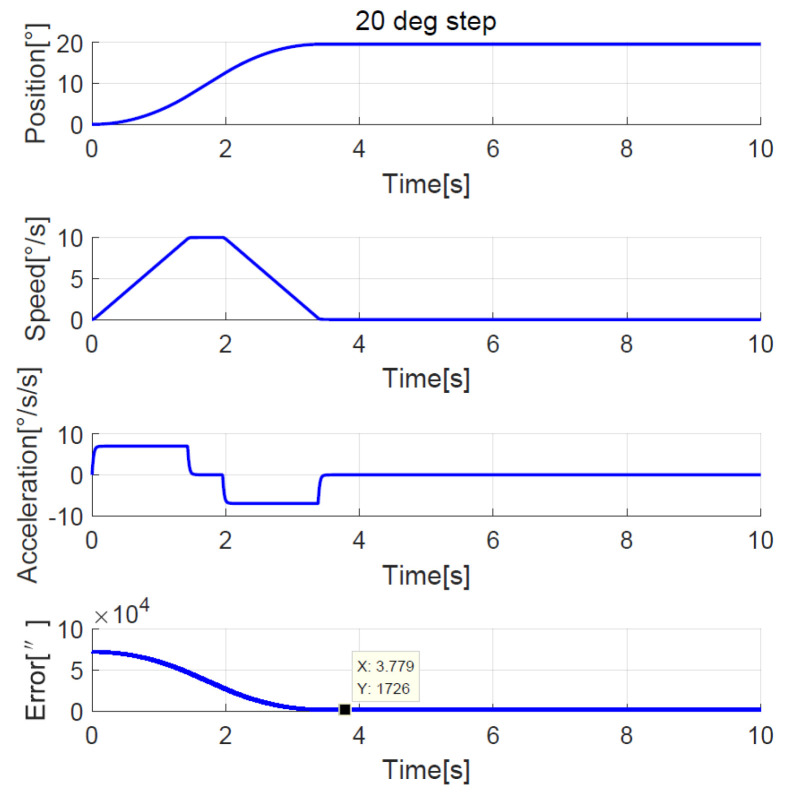
Experimental 20° step response with conventional NTD.

The simulation showed that the transition process was bad without the NTD, so here we only compare the experimental results between the improved NTD and conventional NTD. The parameter settings of the improved NTD are exactly the same as the simulation. The conventional NTD had the same speed factor r as the improved NTD but without considering the speed upper bound allowed by the telescope, its maximum value of the speed is closely related to the step set value p0, that is, $V_{maxNTD}=\sqrt{p_{0}r}$. When the ADRC + NDOB is adopted and the transition process is planned by the improved NTD and conventional NTD, respectively, the improved NTD’s step response curves of 1.24° FOV and 20° are shown in Figure 22 and Figure 23, respectively. The conventional NTD’s step response curves of 1.24° FOV and 20° are shown in Figure 24 and Figure 25, respectively. It can be seen from Figure 22 and Figure 23 that the improved NTD’s results are almost consistent with the simulation, which verifies the effectiveness of the improved NTD in achieving a fast and smooth transition process. In Figure 24, the conventional NTD was not affected by speed saturation when a small step response such as 1.24° FOV was used because $V_{maxNTD}=\sqrt{8.68}=2.94<\Omega_{max}$. But when a 20° step response is used, as in Figure 25, since $V_{maxNTD}=\sqrt{140}=11.8>\Omega_{max}$, the planned speed and position do not match, resulting in the final steady-state error of about 1726 arc-seconds.

The telescope servo has a determined drive capacity, for safety and practical use. It is thus reasonable and necessary to design an improved NTD that takes into account the maximum acceleration and speed. The experimental results show that the improved NTD has better performance than the conventional NTD, especially when tracking large position steps.

## 5. Conclusions

To address the issues facing a 2.5-m-wide field survey telescope suffering from limited control bandwidth and increasing disturbances, a novel cascade anti-disturbances structure (NCADS) with linear active disturbance rejection control (LADRC) and a nonlinear disturbance observer (NDOB) was proposed in this study. The NDOB feedforward compensated for dominant disturbances on the inner current loop, and the LESO of the ADRC rejected the residual disturbance on the outer speed loop, which lightened the burden of the ADRC. Compared with conventional methods such as PI, ADRC, and PI + NDOB, the proposed NCADS method has stronger ability to reject disturbances from friction, cogging torque, and wind load, thus achieving higher tracking precision. A parameter-tuning method based on bandwidth was presented, which makes it easy for engineering applications.

The improved NTD with the speed and acceleration limit of the telescope was proposed to plan the transition process, which does not need to tune other parameters and made the telescope operation faster and smoother.

The simulation and experimental results of the 2.5-m-wide field survey telescope showed that the proposed method has better performance in terms of disturbance rejection, tracking precision, and transition process compared to the conventional methods. There is no need to add an acceleration sensor or change the classical three-closed-loop control structure.

The NCADS method can effectively enhance robustness against disturbances under limited control bandwidth, and the improved NTD is feasible method to plan transition process for servo system that knows the drive capability. We believe that the proposed method has the potential to improve the performance of large telescopes’ servo systems.

The simulations verified the effectiveness of the proposed method for suppressing wind disturbance, but it is necessary to conduct an outfield wind disturbance experiment next. In order to further improve the control efficiency, nonlinear active disturbance rejection control will be studied in the future.

## Figures and Tables

**Table 1:** Servo system parameters.

Parameters	Value
Synchronous inductance $L_{q}$/mH	23.75
Stator phase resistance $R_{s}/\Omega$	2.45
Number of pole pairs $n_{p}$	45
Number of slots in stator *Z*	270
The torque coefficient $K_{t}/\left(N\cdot m/A\right)$	118
The moment of inertia $J/(\mathrm{kg}\cdot m^{2})$	7100
Coefficient of viscous friction $B/(N\cdot m\cdot s\cdot {\mathrm{rad}}^{-1})$	30
Bus voltage $V_{Bus}$/V	60
Limited current $I_{qm}$/A	10

**Table 2:** Friction parameters.

Parameters	Value
Coefficient of viscous friction $B/(N\cdot m\cdot s\cdot {\mathrm{rad}}^{-1})$	30.0
Coulomb friction $F_{c}/\left(N\cdot m\right)$	67.0
Static friction $F_{s}/\left(N\cdot m\right)$	140.0
Stribeck speed $v_{s}/\left(\mathrm{rad}/s\right)$	0.0004

**Table 3:** Comparison of anti-disturbance performance under sudden wind load when the steady-state velocity is 0.01°/s.

Controller Type	Speed Fluctuation/(°/s)	Adjustment Time/(s)
PI	0.0649	/
ADRC	0.0679	0.216
PI + NDOB	0.0255	0.129
ADRC + NDOB	0.0219	0.113

## Data Availability

Not applicable.
